# Complexation of 26-Mer Amylose with Egg Yolk Lipids with Different Numbers of Tails Using a Molecular Dynamics Simulation

**DOI:** 10.3390/foods10102355

**Published:** 2021-10-03

**Authors:** Shangyuan Sang, Xueming Xu, Xiao Zhu, Ganesan Narsimhan

**Affiliations:** 1 College of Food and Pharmaceutical Sciences, Ningbo University, 169 Qixing South Road, Ningbo 315800, China; shangyuan.sang@foxmail.com; 2 The State Key Laboratory of Food Science and Technology, School of Food Science and Technology, Synergetic Innovation Center of Food Safety and Nutrition, Jiangnan University, 1800 Lihu Avenue, Wuxi 214122, China; xmxu@jiangnan.edu.cn; 3 Department of Agricultural and Biological Engineering, Purdue University, West Lafayette, IN 47907, USA; zhu472@purdue.edu; 4 Research Computing, RCAC, Purdue University, West Lafayette, IN 47907, USA

**Keywords:** amylose-lipid complex, molecular dynamics, triglyceride, POPC, cholesterol

## Abstract

A molecular dynamics simulation of mixtures of 26-mer amylose with three different egg yolk lipids, namely, cholesterol, triglyceride and 1-palmitoyl-2-oleoyl-*sn*-glycero-3-phosphocholine (POPC), demonstrated the formation of a stable complex. The 26-mer amylose fluctuated between a coiled and an extended helical conformation. The complex was a V-type amylose complex, with the hydrophobic tail of the lipids being inside the hydrophobic helical cavity of the amylose. The number of glucose units per turn was six for the two helical regions of the amylose-POPC complex and the palmitoyl tail region of the amylose-triglyceride complex. This value was eight for the cholesterol and the two-tail helical region in the amylose-triglyceride complex. Two tails of the POPC were in two different hydrophobic helical regions of the 26-mer amylose, whereas the palmitoyl tail of the triglyceride lay in one hydrophobic helical region and the linoleoyl and oleoyl tails both lay in another helical region, and the cross-sectional area of the latter was larger than the former to accommodate the two tails. The radii of the gyration of the complex were lower for all three cases compared to that of one single amylose. In addition, the stability of the complexes was ranked in the following order: POPC < cholesterol < triglyceride, with their average binding energy being −97.83, −134.09, and −198.35 kJ/mol, respectively.

## 1. Introduction

The amylose component of starch can form complexes, known as V-amylose, with amphiphilic or hydrophobic ligands. The V-amylose complexes are single, left-handed helices that are arranged as crystalline and amorphous lamellae, which may form distinct nano- or micron-scale structures. These complexes are used in a variety of applications. V-amylose has potential as a biomaterial for the nanoencapsulation of sensitive bioactive and flavor ingredients. It also can be used to modify the rheological behavior of starch-containing products, as well as to retard starch retrogradation and postprandial hyperglycemia in diabetics. A detailed review delineating the various aspects of V-amylose’s structure, methods of preparation, factors that affect its formation, and the significance and potential applications of V-amylose complexes can be found elsewhere [1]. The amylose-lipid complex (ALC) resists digestion by human pancreatic amylase in the small intestine and thus reaches the colon, where it is fermented by gut microbes. Since ALC exhibits physiological effects similar to those of dietary fibers, they are of great interest in the food industry. The consumption of ALC has been shown to reduce blood glucose levels in humans and retard the proliferation of colon cancer in rats. Other benefits of ALC include its use as a fat replacement in food [2] and as a carrier for the delivery of bioactive compounds through their encapsulation [3].

Fatty acids complex with V-amylose form a helix with the hydrophobic chain located either fully or partly inside the helix along the helix axis, and with the hydrophilic carboxyl group located outside [4,5,6]. The driving force behind this complexation is the tendency of amylose to minimize interactions with water [7]. The helix cavity provides binding sites for the hydrophobic chain [8]. The helix is found to have six to eight glucosyl residues per turn [9], with its diameter being dependent on the size of the fatty acid [10]. Differential scanning calorimetry studies of complexes indicated that complexation is a reversible process [11]. Van der Waals and hydrophobic forces between the methylene groups of lipid and five-carbon hydrogen in glucose are believed to stabilize the complex [11]. A molecular dynamics simulation of an amylose linoleic acid mixture demonstrated the formation of the complex through a lower root mean square distance, a higher number of hydrogen bonds within the helical cavity and a higher level of amylose–water interaction energy [12]. The amylose-lipid complexation resulted in a change from an extended to a helical arrangement in the amylose conformation [13].

Amylose-lipid complexes can be subdivided into a less ordered type I and semi-crystalline type II [14]. The complex formation by lipids of various chain lengths with wheat and bombara starch in pasting cells indicated a decrease in final viscosity, with a corresponding increase in final viscosity in the presence of lipids [15,16]. The maximum complex formation is found to occur at different lipid concentrations for different chain lengths [16,17]. At sufficiently high concentrations, however, lipids tend to self-associate rather than form a complex [16]. Complex formation is achieved by shear-less low moisture heating [18,19], enzymatic methods or thermo-mechanical methods. The yield and crystallinity of starch-lipid complex depend on the amylose content of the starch variety, with higher amylopectin starches tending to form fewer or no complexes [20]. ALCs can be produced in the laboratory from pure compounds; lipids (mostly fatty acids or monoacyl glycerol) are added to starch and heat-processed in excess water by extrusion cooking or steam-jet cooking to produce the starch-lipid complexes [3]. They are classified as resistant starch types III or V. The minimum amylose DP (degree of polymerization) required for the complexation of fatty acids varies between 20 to 40 glucosyl residues and is about the chain length that can accommodate two fatty acids [21]. At a pH less than pK, short-chain fatty acids tend to make complexes more readily than long-chain fatty acids and vice versa [22]. Complex formation is retarded by starch acetylation [23]. Complexation results in a decrease in enzymatic hydrolysis and, therefore, reduced digestibility [24]. Complex formation increases the starch gelatinization temperature [25] and reduces the swelling capacity by reducing the entry of water molecules into starch granules [26]. Complexation competes with retrogradation, thereby slowing the recrystallization process [27,28].

In a previous study, we investigated the effect of three egg yolk lipids (cholesterol, triglyceride and POPC) on the structure and properties of wheat starch during steamed bread-making (below 100 °C) [29]. However, for heat-processed cereals at high temperatures (above 100 °C) and pressures, such as baking, extrusion cooking and steam jet cooking, the three yolk lipids can dissociate from lipid particles so as to become available for interaction with amylose in egg-containing cereal foods, such as cake and bread. In the current study, we employed a molecular dynamics (MD) simulation to investigate complex formation between a 26-mer amylose fragment and these egg yolk lipids with differing numbers of tails, of different lengths and degrees of unsaturation. The root mean square deviation (RMSD), radius of gyration, potential energy of interaction and binding energy of the different complexes were evaluated in order to characterize the effect of lipid tails on complexation.

## 2. Simulation Methods

A segment of amylose chain consisting of 26 glucose units (26-mer) was employed in all the simulations. The initial structure of the 26-mer amylose was set up using a GLYCAM server (http://www.glycam.org) (accessed on 2 January 2020), as described elsewhere [13]. Briefly, the simulation was carried out for 100 ns after the minimization of the internal energy of the chain using steepest descent [30] to obtain the initial structure. The initial structure of the lipids (cholesterol, POPC and triglyceride) was set up using CHARMM-GUI, as described elsewhere [31]. The initial distance between the center of mass of the 26-mer amylose and center of mass of the lipid was maintained at 2 nm. The 26-mer amylose and the lipid were then placed in a periodic cubic box so that the distance between its atoms and the edge of the box was at least 1 nm (cutoff distance). The box side length was 11.6265 nm for cholesterol and triglyceride, whereas it was 11.0265 nm and 10.6265 nm for POPC and one amylose respectively. The water molecules represented by TIP3P potential were subsequently placed in the box. For the amylose-cholesterol system, the periodic box contained 51,493 water molecules, whereas this number was 43,296 and 51,462 for amylose-POPC and amylose-triglyceride complex respectively. The simulation was also carried out for one 26-mer amylose only; in this case, 38,877 water molecules were employed. The system size for each simulation is summarized in Table 1. This difference in the number of water molecules was due to the difference in the water-accessible volume within the box (of the same dimensions) because of the difference in the molecular volume of the lipids.

The simulation was then carried out for 900 ns using GROMACS software. A CHARMM36 force field was employed for the 26-mer amylose and the lipids in all the simulations. A steepest descent algorithm was employed to minimize the configurational energy of the system [32], with the criteria being either a maximum of 50,000 steps or a maximum force of less than 100 kJ/mol/nm. The system was then equilibrated at a constant volume for 1 ns, which was then followed by heating the system at constant pressure to 300 K in 2 ns. During heating, the backbones of the 26-mer amylose and the lipid were constrained with a spring constant of 1000 kJ/mol/nm. Lennard Jones potential with a cutoff at 1.2 nm and full electrostatics with particle-mesh Ewald summation were employed. The covalent bonds were constrained by a LINCS algorithm with a time step of 2 fs. A production MD simulation was run with a timestep of 2 fs up to 900 ns. A leap frog algorithm for the integration of Newton’s equation was used. The solvation, potential, van der Waals, electrostatic and binding energies of the complex were evaluated for every 0.5 ns using the g_mmpbsa program (https://rashmikumari.github.io/g_mmpbsa/) (accessed on 2 January 2020), the details of which are given elsewhere [33]. The conformations at different times were visualized using VMD software. The RMSD and radius of gyration of the 26-mer amylose-lipid complex were calculated at different times during the simulation. In these calculations, the structure at 800 ns was employed as the reference. In addition, the RMSD and radius of gyration of the 26-mer amylose only when present as a complex were also calculated from 800 to 900 ns in all the simulations. The time average of these values was calculated within a time interval of 800–900 ns, which was based on the simulation values at every 0.5 ns. The presence of intramolecular H-bonds of 26-mer amylose was evaluated by a criterion in which the maximum distance between the donor and acceptor atoms was 0.35 nm and the angle for the hydrogen-donor-acceptor was below 30° [12]. The number of glucose units per turn was calculated by the subtraction of the residue number at the same position along a helix of the structure at approximately 800 ns simulation in VMD software.

## 3. Results and Discussion

### 3.1. Structure

**Figure 1:**
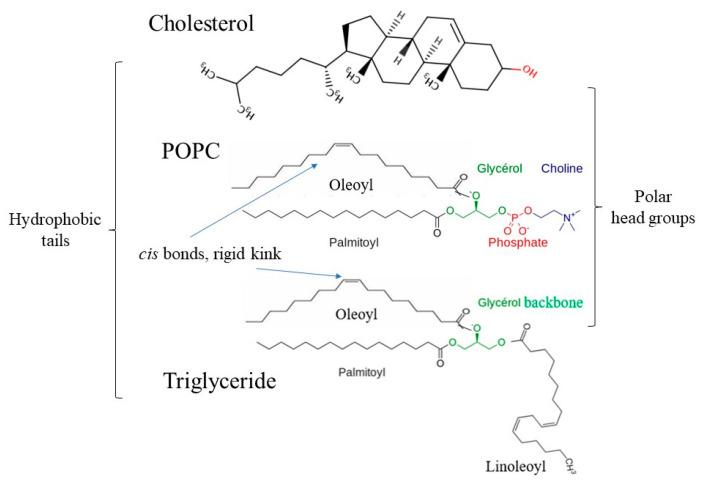
Chemical structure of three egg yolk lipids.

The chemical structures of the three egg yolk lipids are shown in Figure 1. Cholesterol has a hydroxyl head and a hydrophobic tail. POPC has two tails (oleoyl and palmitoyl) and a zwitterionic choline head consisting of a negatively charged phosphate group and a positively charged amine group. Triglyceride has three tails (palmitoyl, oleoyl and linoleoyl groups) and a glycerol backbone. These calculations were performed with part of an amylose molecule consisting of 26 glucose units.

**Figure 2:**
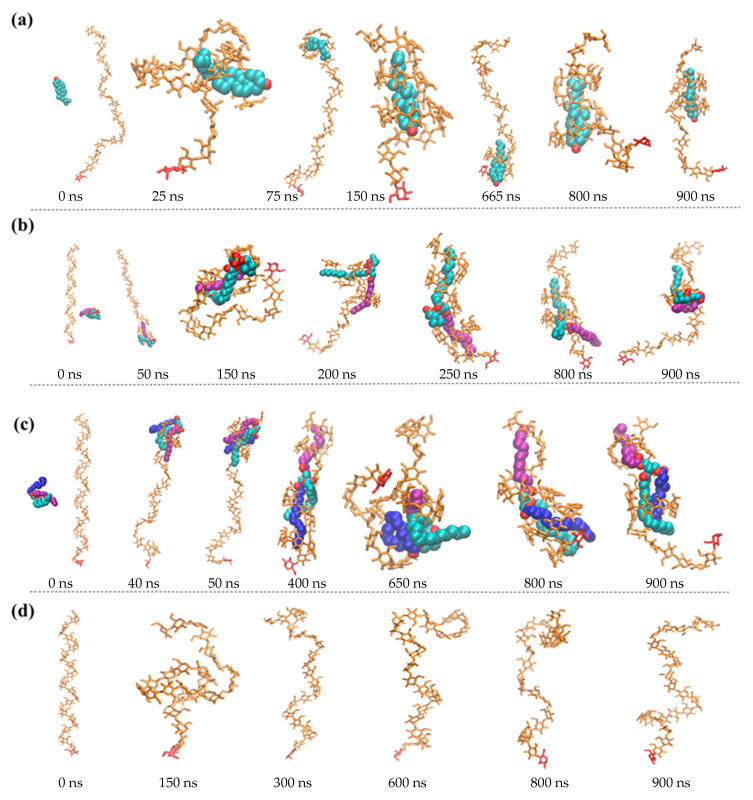
Snapshots of one-amylose and one-amylose-lipid complexes at various times (0–900 ns): amylose-cholesterol complex ((**a**) oxygen atom of cholesterol is red), amylose-POPC complex ((**b**) palmitoyl tail of POPC is in purple, oleoyl tail of POPC is in green), amylose-triglyceride complex ((**c**) linoleoyl tail of triglyceride is in blue) and amylose ((**d**) backbone is in orange and reducing end of 26-mer amylose is in red).

Figure 2a–c show the snapshots of complexation of one amylose molecule with cholesterol, POPC and triglyceride, respectively, at different times. In all the calculations, the initial distance between the center of mass of the amylose and the lipid was kept the same, at 2 nm. As can be seen from Figure 2, the two molecules moved towards each other as time progressed within the first 100 ns because of favorable interaction. In all three cases, the amylose had an extended helical structure [13], which was found to flap, thereby resulting in fluctuations in binding free energy, as is discussed later.

In the case of cholesterol, at 800 ns, the molecule resided inside the helical structure of part of the amylose, with its hydroxyl group protruding outside (Figure 2a). The structure of the complex as obtained by the MD simulation indicated that the cholesterol resided inside the hydrophobic helical cavity, which was consistent with molecular modeling of the complexation of fatty acid with 15-mer amylose [5].

For the interaction between POPC and amylose, the two tails (oleoyl and palmitoyl) of the POPC interacted more strongly with the amylose and one of the two was found to reside inside the helical part of the amylose, with the other interacting with the surface of the helix. The two tails interchanged their positions at different times. The hydrophilic charged head, however, lay outside because of more favorable interactions with the aqueous medium (Figure 2b). The structure of the complex, as obtained by the MD simulation, indicated that the lipids resided inside the hydrophobic helical cavity, which was consistent with a previous molecular dynamics simulation with 13-mer amylose [13]. By contrast, in their simulation of a DPPC-amylose complex, only one of the two hydrophobic tails lay inside the helical cavity. This difference may have been due to the difference in the structure of the tails of the two molecules. In the DPPC, the two tails were both saturated and parallel, whereas in the POPC, the two tails were separated. As a result, steric constraint may have prevented the two tails from being in one hydrophobic cavity in the case of the DPPC. This simulation demonstrates the formation of a complex between two initially separated molecules, as opposed to their simulation, where the complex was already formed initially.

In the case of the triglyceride’s interaction with the amylose, the palmitoyl tail resided inside the helical part of the molecule, whereas both oleoyl and linoleoyl tails together resided inside another helical part of the molecule (Figure 2c). The helical structure of the 26-mer amylose was hydrophilic on the outside and hydrophobic on the inside, which was consistent with previously reported results [34]. Figure 2d shows how the 26-mer amylose fluctuated between a coiled and an extended helical conformation [35]. The number of intramolecular H-bonds of amylose alone was 10, less than the corresponding values for the complexed helical amyloses with cholesterol (18), POPC (16) and triglyceride (21) (Table 1).

In addition, the fine helical structure of amylose in complex—number of glucose units per turn, pitch, and the size of helical cavity are summarized in Table 2. The number of glucose units per turn was eight for the amylose-cholesterol complex, whereas this number was six for the POPC (each tail) and eight (oleoyl and linoleoyl tails) and six (palmitoyl tail) for the triglyceride. As both oleoyl and linoleoyl tails for the triglyceride resided inside the same helical part of the amylose molecule during 800–900 ns of simulation (Figure 2c), the values were the same, as can be seen in the last column of Table 2. The external diameter of 1.08 to 1.744 nm, with an internal diameter of 0.696 to 1.207 nm for our simulation, compares favorably with the reported external diameter of 1.3 to 1.37 nm and the internal diameter of 0.5 to 0.54 nm for Vh amylose with the inclusion of iodine [34,36] or aliphatic ketone complex. This difference can be attributed to the space required to accommodate the molecules within the cavity, because the helical cavity size in the two-tail region was larger than that in the one-tail region. Understandably, the accommodation of two tails requires a larger cavity and, hence, a larger number of glucose units per turn. For cholesterol, however, a larger cavity is required because of its larger cross-sectional area. Similar behavior in a larger cavity was observed for the inclusion of more voluminous ligands inside amylose [37]. Complex formation by starch with lactones has been observed through X-ray crystallography [38], which also indicated a V-type helix complex. The number of glucose units per turn obtained in our simulations compares favorably with the values of seven or six for V-type complexes, as suggested by some researchers [5,39,40,41,42].

### 3.2. RMSD, Radium of Gyration, and Number of H-Bonds

**Figure 3:**
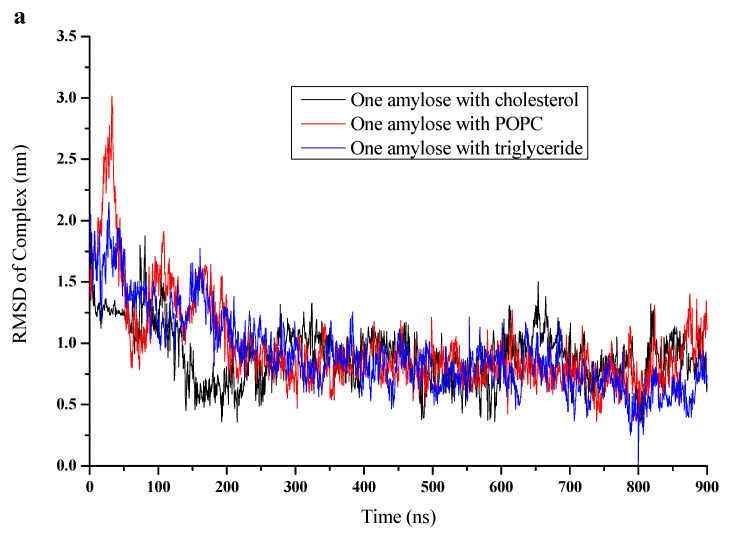

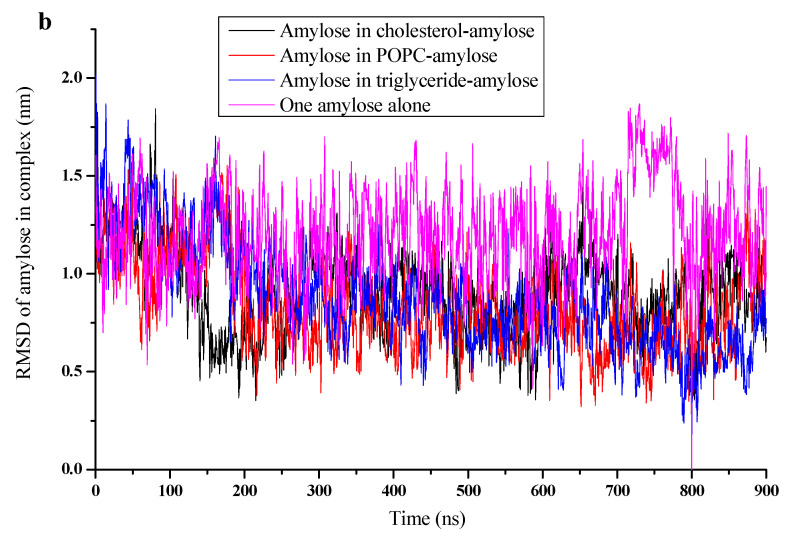
RMSD (nm) versus time (0 to 900 ns) for (**a**) complex of amylose-cholesterol (black), amylose-POPC (red) and amylose-triglyceride (blue); (**b**) for amylose in amylose-cholesterol complex (black), amylose-POPC complex (red), amylose-triglyceride complex (blue) and amylose alone (pink).

Figure 3a shows the evolution of the RMSD for the one-amylose-lipid complex for cholesterol, POPC and triglyceride respectively. The RMSD for the one-amylose-cholesterol complex decreased faster within the first 200 ns and stabilized at much larger times (up to 900 ns). For the one-amylose-POPC and the one-amylose-triglyceride complexes, however, the RMSD decreased more gradually up to 300 ns and stabilized at longer times. Furthermore, the stabilized average RMSD value for the amylose-cholesterol complex was around 0.5 nm compared to the value of 1 nm for the other two complexes at 200 ns. This indicates that the more stable complexation of amylose with cholesterol occurred at shorter times. The RMSD values at longer times seemed to fluctuate between 0.6 to 1.2 nm, possibly because of flapping of the long amylose tail that was not interacting with the lipid. The RMSD values averaged over the last 100 ns, as given in Table 1, indicated that the one-amylose-triglyceride complex was the most stable, followed by the POPC and cholesterol complexes.

In order to ascertain the stability of amylose conformation in the complex, the RMSD values that were based on the position of the amylose molecule only are shown in Figure 3b. The RMSD value averages over the last 100 ns (800 to 900 ns) for different complexes are given in Table 1. The average value was found to be the lowest for the triglyceride, followed by the POPC and the cholesterol. Consequently, triglyceride stabilized one amylose molecule the most, followed by POPC and cholesterol, which followed the same order as the stability of the complex as discussed above.

**Figure 4:**
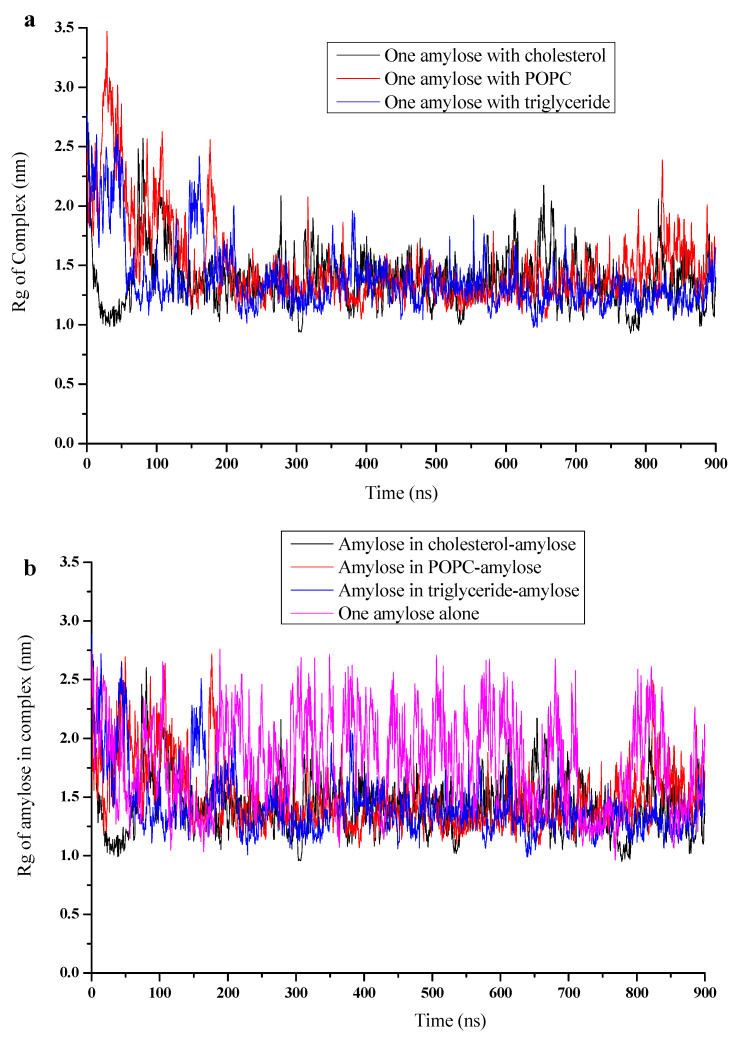
Radius of gyration (Rg, nm) versus time (0 to 900 ns) for (**a**) complex of amylose-cholesterol (black), amylose-POPC (red) and amylose-triglyceride (blue); (**b**) for amylose in amylose-cholesterol complex (black), amylose- POPC complex (red), amylose-triglyceride complex (blue) and amylose alone (pink).

The evolution of the radius of gyration for the amylose-lipid complexes for amylose-cholesterol, amylose-POPC and amylose-triglyceride are shown in Figure 4. The radius of gyration seemed to stabilize after about 200 ns, although, as pointed out above, the fluctuation was pronounced because of flapping of the part of the amylose that did not interact with the lipid. The radius of gyration values averages over 800–900 ns for the three complexes are given in Table 1. The average radius of gyration was the smallest, with a value of 1.25 nm, for the amylose-triglyceride complex and the largest, with a value of 1.55 nm, for the amylose-POPC complex.

Since their simulations were performed for 900 ns, amylose showed some fluctuations between a coiled and extended helix. As shown by the results, the number of intramolecular H-bonds in amylose alone was 10, less than that of the complexed helical amyloses with cholesterol (18), POPC (16) and triglyceride (21) (Table 1). Interestingly, the number of intramolecular H-bonds was found to be the lowest for amylose alone and correlated inversely with the radius of gyration (Table 1). A comparison of these values with that of the amylose alone indicates that the complexed amylose was more densely packed and more stable than amylose alone.

### 3.3. Binding Energy

**Figure 5:**
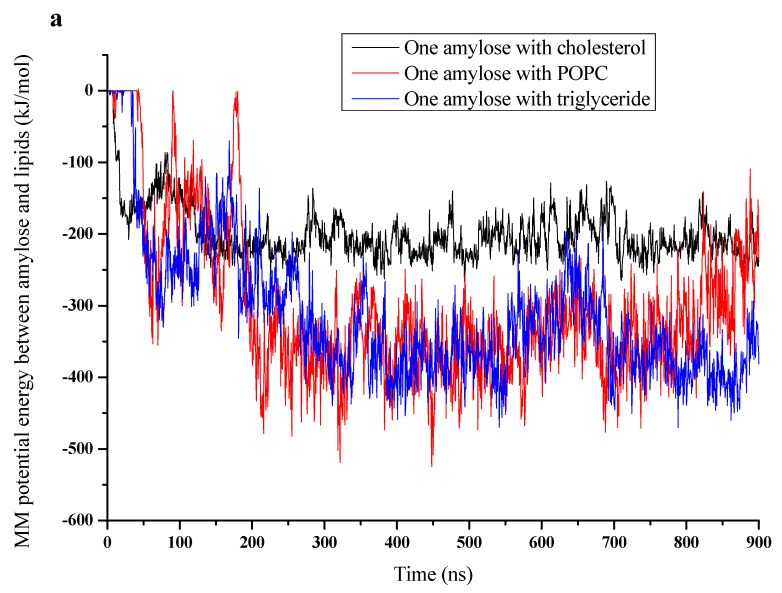

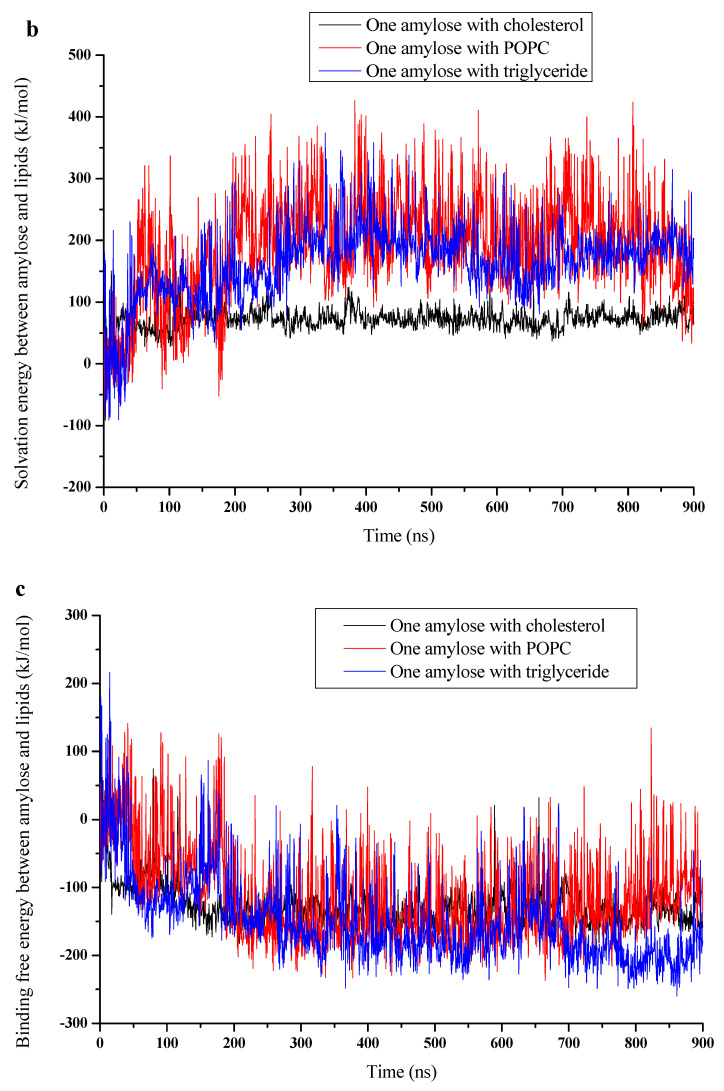
MM potential energy (**a**), solvation energy (**b**) and binding free energy (**c**) for amylose-cholesterol complex (black line), amylose-POPC complex (red line) and amylose-triglyceride complex (blue line).

Figure 5a shows the variation of potential energy with time for the three complexes. Interestingly, the potential energy was highest (least negative) for cholesterol, followed by POPC and triglyceride. This correlates well with the number of atoms in the three lipids. The average values are given in Table 3. The average values of contributions from van der Waals and electrostatic interactions are also given in Table 3. The contribution of electrostatic interaction was highest (15.7%, electrostatic/MM potential energy) for the POPC complex and much lower for the other two. This is believed to be due to the higher charge density of the hydrophilic head in the former compared to the latter. The evolution of solvation energy of the amylose-lipid complex (Figure 5b) and the values averaged over 800–900 ns for the three lipids (Table 3) are given. The solvation energy was positive for all three cases, with the cholesterol complex having the least, followed by POPC and triglyceride. The value for the cholesterol complex was almost half that of the other two.

Binding free energy is the sum of potential and solvation free energies. Figure 5c shows the evolution of binding free energy between amylose and lipid molecules for cholesterol, POPC and triglyceride, respectively. In these calculations, the initial distance of separation between the centers of mass of the amylose and the lipid was 2 nm. As expected, the binding free energy decreased and became negative (favorable) at short times (within 200 ns) and stabilized thereafter. The long time average (between 800 and 900 ns) of the binding free energy, as given in Table 3, indicates that the binding energy was lowest for amylose-triglyceride complex followed by cholesterol and POPC. This was consistent with the results reported by Chao et al., that the amylose-tripalmitate glycerol complex was the most stable among dipalmitate and monopalmitate glycerol complexes as its thermal transition temperature was the highest during heating and reheating [43]. As pointed out above, the large fluctuations in binding free energy for all three complexes is believed to have been due to the flapping (fluctuation) of uncomplexed tail fragments of amylose within the complexes.

Our results indicated that the complex formed with the triglyceride with three tails was the most stable, followed by the POPC with two tails and the single-tail cholesterol. This is in accordance with observations that the stability of complexes increases with more hydrophobic interactions between lipids (fatty acids, surfactants, tripalmitate glycerol) and amylose [43,44,45]. It is believed that this non-polar interaction energy is more negative for the complex compared to amylose only in order to stabilize the complex. The negative value of the binding free energy (with respect to the reference of the 26-mer amylose) indicates that the 26-mer amylose-lipid complex was more stable than amylose alone. Van der Waals interaction energy in the complex was influenced by the number of hydrophobic groups that interacted with amylose within the helical cavity. Understandably, the van der Waals interaction was the largest for the amylose-triglyceride complex, followed by the POPC complex and then the cholesterol complex. The number of hydrophobic groups was largest for the three-tail lipid, followed by the two-tail lipid and the single-tail lipid [34,43,46]. As already pointed out above, the electrostatic interaction energy was much larger for the 26-mer amylose-POPC complex than for the other two complexes because of the much higher charge density in the former compared to the latter.

## 4. Conclusions

An MD simulation of a mixture of 26-mer amylose with three different lipids, namely, cholesterol, triglyceride and POPC, was performed to characterize the effect of the length of tails on complex formation. The RMSD, radius of gyration, potential energy of interaction and binding free energy were evaluated. The 26-mer amylose was found to fluctuate between coiled and extended helical conformations. All three lipids formed a complex with amylose. Two tails of the POPC were in two different hydrophobic helical regions of the 26-mer amylose; in case of the triglycerides, palmitoyl tail lay in one hydrophobic helical region, whereas linoleoyl and oleoyl tails both lay in another helical region. Understandably, the cross-sectional area of the latter was larger than the former in order to accommodate two tails. The complex was a V-type amylose complex; the hydrophobic tail of the lipid was found to lie inside the hydrophobic helical core of the amylose. The number of glucose units per turn was six for the two helical regions of the amylose-POPC complex and the palmitoyl tail region of the amylose-triglyceride complex. This value was eight for the cholesterol and the two-tail helical region of the amylose-triglyceride complex. The binding energy of the three complexes was negative, indicating that they were stable.

## Figures and Tables

**Table 1:** Cubic box side length, water number and time average (800–900 ns) conformation parameters of amylose-lipid complexes and one amylose alone.

Simulations	Box Side Length (Nm)	Water Molecule Number	RMSD of Complex (Nm)	RMSD Of Amylose (Nm)	Rg of Complex (Nm)	Rg of Amylose (Nm)	NO. of H-Bonds
Cholesterol	11.6265	51,493	0.879	0.888	1.37	1.41	18
POPC	11.0265	43,296	0.862	0.829	1.55	1.6	16
Triglyceride	11.6265	51,462	0.593	0.614	1.25	1.3	21
Amylose alone	10.6265	38,877	-	1.19	-	1.75	10

RMSD, root mean square deviation; Rg, radius of gyration; POPC, 2-oleoyl-1-palmitoyl-*sn*-glycero-3-phosphocholine; NO. of H-bonds, the number of intramolecular hydrogen bonds in amylose.

**Table 2:** Conformation parameters of helical regions of amylose-lipid complexes.

	Helical Regions	Cholesterol	POPC		Triglyceride	
Parameters		Hydrophobic Tail	Palmitoyl Tail	Oleoyl Tail	Palmitoyl Tail	Oleoyl and Linoleoyl Tails
NO. of glucose units per turn		8	6	6	6	8
Maximum NO. of glucose units in each helical region		16	13	16	13	18
Pitch of helical regions (nm)		0.992	0.877	0.789	0.774	1.051
Inner diameter of helical cavity (nm)		0.927	0.696	0.871	0.802	1.207
Outer diameter of helical cavity (nm)		1.744	1.080	1.249	1.314	1.530

**Table 3:** Time average (from 800 to 900 ns) energy parameters for amylose-lipid complexes.

	Parameters	Van Der Waals (kJ/mol)	Electrostatic (kJ/mol)	MM Potential (kJ/mol)	Solvation (kJ/mol)	Binding Free Energy (kJ/mol)
Simulations						
Cholesterol		−207.62	−3.88	−211.5	76.6	−134.89
POPC		−231.97	−43.29	−275.26	178.23	−97.03
Triglyceride		−363.13	−22.83	−385.96	187.61	−198.35

MM, molecular mechanics; POPC, 2-oleoyl-1-palmitoyl-*sn*-glycero-3-phosphocholine.

## Abbreviations

ALC	amylose-lipid complex
DP	degree of polymerization
DPPC	dipalmitoylphosphatidylcholine
MD	molecular dynamics
POPC	1-palmitoyl-2-oleoyl-*sn*-glycero-3-phosphocholine
RMSD	root mean square deviation
